# Novel Fluorometric Assay of Antiglycation Activity Based on Methylglyoxal-Induced Protein Carbonylation

**DOI:** 10.3390/antiox12122030

**Published:** 2023-11-22

**Authors:** Shin Koike, Yuna Saito, Yuki Ogasawara

**Affiliations:** Department of Analytical Biochemistry, Meiji Pharmaceutical University, 2-522-1 Noshio, Kiyose, Tokyo 204-8588, Japan; skoike@my-pharm.ac.jp (S.K.); y191129@std.my-pharm.ac.jp (Y.S.)

**Keywords:** advanced glycation end products, antiglycation activity, argpyrimidine, bovine serum albumin, carbonyl stress, fluorometric assay, hen egg lysozyme, methylglyoxal, oxidative stress

## Abstract

Advanced glycation end products (AGEs), which can have multiple structures, are formed at the sites where the carbonyl groups of reducing sugars bind to the free amino groups of proteins through the Maillard reaction. Some AGE structures exhibit fluorescence, and this fluorescence has been used to measure the formation and quantitative changes in carbonylated proteins. Recently, fluorescent AGEs have also been used as an index for the evaluation of compounds that inhibit protein glycation. However, the systems used to generate fluorescent AGEs from the reaction of reducing sugars and proteins used for the evaluation of antiglycation activity have not been determined through appropriate research; thus, problems remain regarding sensitivity, quantification, and precision. In the present study, using methylglyoxal (MGO), a reactive carbonyl compound to induce glycation, a comparative analysis of the mechanisms of formation of fluorescent substances from several types of proteins was conducted. The analysis identified hen egg lysozyme (HEL) as a protein that produces stronger fluorescent AGEs faster in the Maillard reaction with MGO. It was also found that the AGE structure produced in MGO-induced in HEL was argpyrimidine. By optimizing the reaction system, we developed a new evaluation method for compounds with antiglycation activity and established an efficient evaluation method (HEL–MGO assay) with greater sensitivity and accuracy than the conventional method, which requires high concentrations of bovine serum albumin and glucose. Furthermore, when compounds known to inhibit glycation were evaluated using this method, their antiglycation activities were clearly and significantly measured, demonstrating the practicality of this method.

## 1. Introduction

AGEs are heterogeneous compounds derived from the nonenzymatic Maillard reaction of reducing sugars with the free amino groups in proteins, nucleic acids, and lipids [1]. Protein carbonylation is a type of protein oxidation that can be promoted by reactive oxygen species. It usually refers to a process that forms reactive dicarbonyl compounds, such as glyoxal, methylglyoxal (MGO), and deoxyglucosones [2]. Numerous studies have implicated protein glycation (carbonylation) in the etiology of atherosclerosis, diabetes, end-stage renal disease, and neurodegenerative disease [3].

Various strategies have been considered to inhibit the glycation reaction as the complex processes that lead to AGE formation involve several pathways, intermediates, and end products. However, amino groups (mostly lysine or arginine) make proteins more susceptible to glycation-induced oxidative modification, resulting in structural alterations and functional changes [4,5]. It has been well documented that the inhibition of nonenzymatic glycosylation reactions can be a beneficial approach to counter the secondary complications of chronic diabetes. Many pharmacological approaches, together with the use of inhibitors, have been established to constrain the glycation reaction and the consequent glycation signaling pathway [6,7,8,9]. Of the natural and chemically synthesized agents that have been suggested as glycation inhibitors, several have either only been studied in vitro or are associated with unwanted complications [7,8,9]. Given the side effects of synthetic compounds, there is a need for alternative approaches that can prevent nonenzymatic glycation [6,7,8]. Therefore, as part of the development of therapeutic agents for AGE-associated diseases, it is critical to find antiglycation compounds with fewer side effects; consequently, a simple and sensitive method for evaluating antiglycation activity will be useful for this research.

Several colorimetric and fluorometric methods are available to determine various parameters that are indicators of AGE production, such as the modification rate of lysine and arginine side chains, fructosamine content, aggregation state of the modified proteins, and AGE-specific fluorescence [10]. Analytical methods have also been employed for the direct assessment of antiglycation capacity [11,12]. In contrast, the antiglycation activity of various compounds was measured by evaluating their cytoprotective effect [13,14,15,16] or their ability to decrease the protein carbonyl content [13,14,15,16] in cultured cells. In addition, several substances have been reported to interfere with nonenzymatic glycation by directly trapping the intermediate α-dicarbonyl compounds [17,18,19]. However, there is currently no convenient, sensitive, and accurate first screening method to evaluate antiglycation activity.

Among the many methods for determining antiglycation activity, fluorescence-based methods have been used widely as a simple detection process to examine the formation and inhibitory effects of AGEs. Conventionally, the reaction system for the fluorescence method uses high concentrations (100–500 mM) of glucose and ribose as the reducing sugars and bovine serum albumin (BSA) as the protein to be carbonylated. Generally, fluorescence at approximately 400 nm can be observed after incubation at 37 °C for 2–4 weeks under neutral conditions [20]. However, the optimum conditions are unclear; many studies have reported that they have applied conditions close to those typical in previous studies [21].

Therefore, in the present study, we developed a new, highly sensitive and efficient screening method that is useful for the discovery of compounds that inhibit glycation. Subsequently, we applied this evaluation method to representative compounds known to have antiglycation activity.

## 2. Materials and Methods

### 2.1. Materials

LC–MS grade acetonitrile (Thermo Scientific Chemicals, Waltham, MA, USA) and nonafluorovaleric acid (NFPA) (Tokyo Chemical Industry Co., Ltd., Tokyo, Japan) were used. All aqueous solutions were prepared with pure water produced by a Milli-Q system (Merck Millipore, Tokyo, Japan). BSA, hen egg lysozyme (HEL), horse myoglobin, bovine milk casein, cyanidin 3-*O*-glucoside chloride, gallic acid glycyrrhizic acid, and cysteamine hydrochloride were purchased from Fujifilm-Wako Chemical (Osaka, Japan). Aminoguanidine hydrochloride (AG), Nα-tertiary butyloxycarbonyl (t-BOC)-arginine, human serum albumin, and human immunoglobulin were purchased from Sigma-Aldrich Chemical (St. Louis, MO, USA). Purified MGO was prepared by a previously reported method [22]. Argpyrimidine (ARP) was synthesized by a previously reported method [23] using MGO and Nα-t-BOC-arginine.

### 2.2. Identification of Argpyrimidine by LC–MS/MS

ARP for protein hydrolysis was prepared [24] and analyzed using LC–MS/MS [25] as described previously, with minor modifications. The LC–MS/MS system consisted of two LC-30AD pumps, a SIL-30AC auto-sampler, a CTO-20A column oven, a CBM-20A system controller (Shimadzu, Kyoto, Japan), and a triple quadrupole mass spectrometer LCMS-8040 (Shimadzu, Kyoto, Japan). A reverse-phase column (ODS-4, 3.0 mm × 150 mm, GL Sciences, Tokyo, Japan) was used for the chromatographic separation. The chromatogram acquisition, detection of mass spectral peaks, and waveform processing were performed using LabSolutions Insight software (Shimadzu, Version 2). The binary mobile phase consisted of elution buffer A (5 mM NFPA in water) and elution buffer B (5 mM NFPA in acetonitrile). Separation was performed using the following gradient from B to A, at a flow rate of 0.4 mL/min: 0–15 min, 15–45% B; 15–16 min, 45–90% B; 16–18 min, 90% B; 18–19 min, 90–15% B, and 19–25 min, 15% B.

The LC effluent was analyzed by MS/MS using an electrospray ionization interface operating in positive ion mode. The tandem mass spectrometer was tuned in the multiple reaction monitoring (MRM) mode to monitor mass transitions in positive ion mode: ARP; *m*/*z* 255.40 (precursor ion), *m*/*z* 140.20 (product ion).

### 2.3. Detection of Intrinsic Fluorescent AGEs by Fluorescence Spectroscopy

Fluorescence spectra were collected by exciting the samples (BSA–MGO, HEL–MGO) at 200–400 nm and recording the fluorescence emission intensity at 300–500 nm using a fluorescence spectrophotometer (F-2700, HITACHI, Tokyo, Japan).

### 2.4. Western Blotting Analysis

Samples were heated for 5 min at 90 °C in SDS sample buffer with a reducing reagent (Nacalai Tesque, Kyoto, Japan). The proteins were separated by SDS–polyacrylamide gel electrophoresis (15%, 5–20% gradient gel, ATTO, Tokyo, Japan) and transferred to polyvinylidene difluoride (PVDF) membranes (Millipore, MA, USA). Nonspecific binding to the membrane was blocked by using 4% (*w*/*v*) Block Ace (KAC Co., Ltd., Kyoto, Japan). The washed PVDF membranes were then incubated for 1 h at room temperature with a primary anti-PEN (pentosidine) antibody (monoclonal, Tans Genic Inc., Tokyo, Japan) or anti-ARP antibody (monoclonal, NIKKEN SEIL Co., Ltd., Shizuoka, Japan). After extensive washing in TPBS, the blots were incubated at room temperature for 1 h with anti-mouse IgG horseradish peroxidase-conjugated secondary antibodies (Vector, Burlingame, CA, USA). Protein bands were detected using Crescendo Western Reagents (Millipore, Billerica, MA, USA) in a ChemiDoc touch image system (Bio-Rad Laboratories, Tokyo, Japan). Quantification of the results was performed by densitometry using Image Lab Software version 5.2 (Bio-Rad Laboratories, Tokyo, Japan).

### 2.5. BSA–Glucose Assay

The glycated BSA was generated as described previously [26]. In brief, 10 mg/mL BSA was incubated with 50 mM glucose in phosphate-buffered saline (PBS, pH 7.4) in the presence or absence of test compounds at 37 °C for 1 week. Test compounds and glucose were prepared in PBS solution; the mixture without BSA was used as a blank sample in the fluorescence assay. The formation of fluorescent AGEs during albumin glycation was evaluated by monitoring their fluorescence at the excitation wavelength of 370 nm and the emission wavelength of 440 nm using a microplate spectrofluorometer (EnSpire 2300, Perkin Elmer, Tokyo, Japan).

### 2.6. HEL–Methylglyoxal (MGO) Assay

The test compound (0.5 mM) was incubated with MGO (0.5 mM) for 24 h at 37 °C in PBS; this reaction mixture was then incubated with HEL (1.0 mg/mL) or BSA (1.0 mg/mL) for 1 week. After appropriate dilution by PBS, the fluorescence intensity was immediately measured at 390 nm, exciting at 320 nm using a microplate fluorospectrometer. To correct for the fluorescent background, the baseline of the corresponding test solution without protein was subtracted. Antiglycation activity was determined from the decrease in fluorescence intensity. The percentage inhibition of the formation of fluorescent AGEs was calculated from Equation (1).

Inhibition (%) = [(*F*_0_ − *F*)/*F*_0_] × 100 (1)

In this equation, *F*_0_ and *F* are the fluorescence intensities of the sample solution in the absence and presence of test compound, respectively.

### 2.7. Statistical Analysis

The values are presented as the mean ± S.D. Statistical significance of differences was assessed using Dunnett’s multiple comparison test to compare the means of three or more groups. Statistical significance was set at *p* < 0.05.

## 3. Results

**Figure 1:**
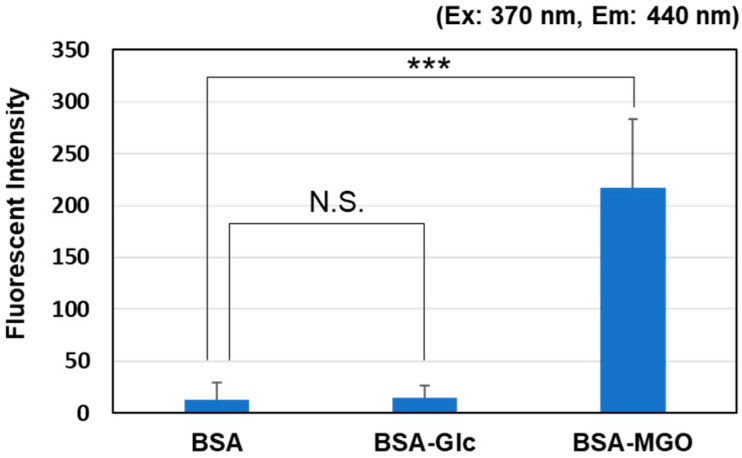
Comparison of intensity changes in BSA fluorescence assay. Fluorescence intensity of BSA solution (in PBS, pH 7.4) measured at an excitation wavelength of 370 nm and emissions wavelength of 440 nm after incubation with glucose (50 mM) or MGO (0.5 mM) at 37 °C for 1 week. *** *p* < 0.001 vs. BSA. N.S.; not significant.

The fluorescence intensity (Ex: 370 nm, Em: 440 nm) of the BSA solution (in PBS, pH 7.4) was measured after incubation with glucose (50 mM) or MGO (0.5 mM) at 37 °C for 1 week using conventional methods. As shown in Figure 1, the reaction between glucose and BSA was slow, and a marked increase in fluorescence intensity was observed when MGO was used. Therefore, to develop a more efficient and sensitive evaluation method, we decided to use methylglyoxal, a product of sugar metabolism, instead of glucose as the component that generates fluorescent AGEs.

**Figure 2:**
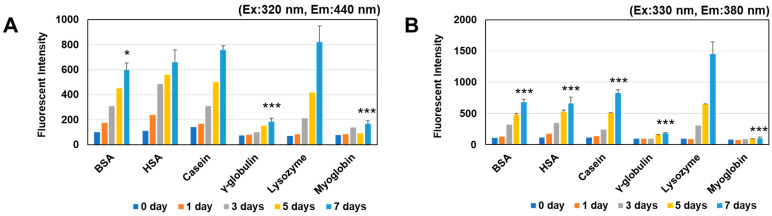
Time courses of fluorescence intensity in the reaction mixture containing various proteins with MGO. The changes in the fluorescence intensity of each solution of protein (1 mg/mL) in the presence of 0.5 mM MGO were observed for 7 days. (**A**) Excitation: 320 nm, Emission: 440 nm, (**B**) Excitation: 330 nm, Emission: 380 nm. * *p* < 0.05, *** *p* < 0.001 vs. Lysozyme.

Next, we selected six proteins known to undergo glycation and observed time-dependent changes in the fluorescence intensity at two wavelengths (440 nm, which is a frequently used wavelength [27], and 380 nm, the optimal wavelength for ARP and PEN [24]. As a result, a marked increase in fluorescence intensity was observed at 440 nm (Figure 2A) and 380 nm (Figure 2B) for BSA, HSA, casein, and HEL reacted with MGO for one week. In a comparison of the two wavelengths, the fluorescence intensities for six proteins obtained at 380 nm was higher than that at 440 nm, and the highest fluorescence intensity was significantly obtained at 380 nm for the reaction of HEL and MGO for a week (Figure 2).

**Figure 3:**
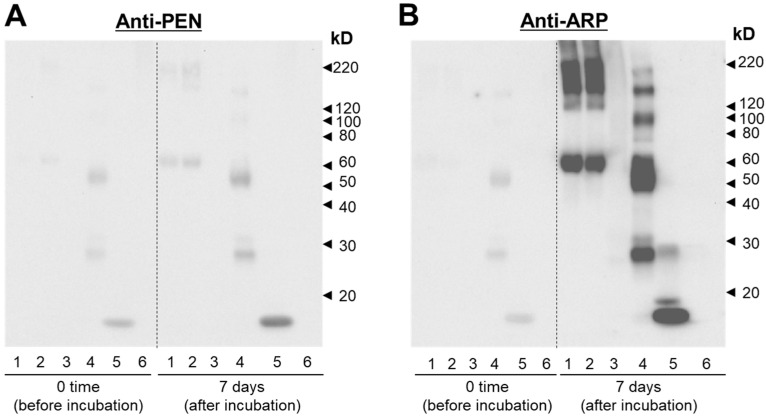
Analysis of quantitative and qualitative changes in fluorescent AGEs by western blotting. (**A**) Western blotting analysis of six proteins using anti-ARP antibodies (left image, 0 time; right image, 7 days after incubation). (**B**) Western blotting analysis of six proteins using anti-PEN antibodies (left image, 0 time; right image, 7 days after incubation). Lane 1: BSA, Lane 2: HSA, Lane 3: Casein, Lane 4: γ-globulin, Lane 5: Lysozyme, Lane 6: Myoglobin.

Based on the result that high fluorescence intensity was obtained around 380 nm, we estimated the formation of ARP and PEN as fluorescent AGEs produced by the reaction of each protein with MGO. Therefore, western blotting was used to examine changes in the formation of ARP and PEN structures in the six protein molecules. As a result, an increase in band intensity detected with anti-ARP antibodies was observed for all proteins except for horse myoglobin (Figure 3B); however, only a small increase in band intensity and multimerization was observed with anti-PEN antibodies (Figure 3A).

To further investigate the structure of the fluorescent AGEs produced in the reaction with MGO, the two proteins were acid hydrolyzed after the aminocarbonyl reaction, and the resulting solution was separated by LC-MS/MS and the peaks detected were analyzed.

**Figure 4:**
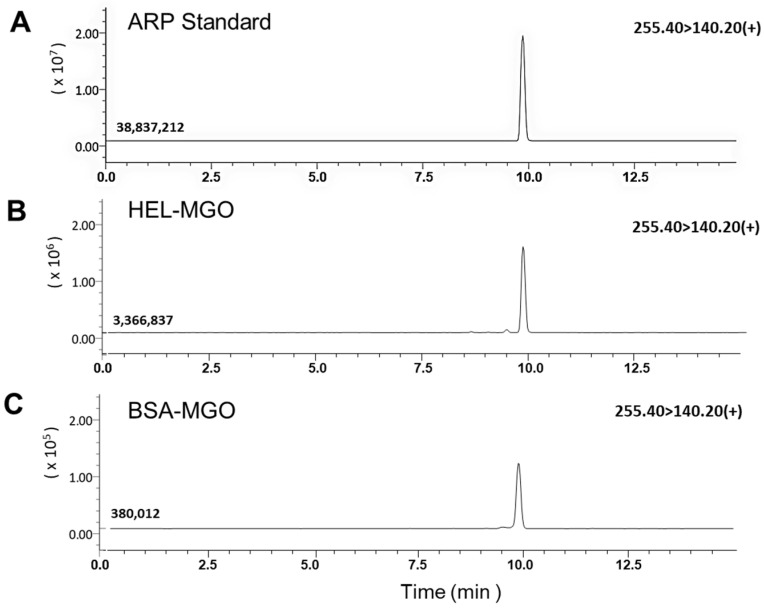
LC–MS/MS analysis of ARP produced in the HEL and BSA after reaction with MGO for 7 days. Each protein solution was acid-hydrolyzed after the reaction, and the mass fragments of the peaks that coincided with the elution time of the free form of standard ARP were compared and analyzed. The tandem mass spectrometer was tuned in the MRM mode to monitor mass transitions in positive ion. ARP; *m*/*z* 255.40 (precursor ion) → 140.20 (product ion). (**A**) ARP standard, (**B**) HEL-MGO, (**C**) BSA-MGO.

As shown in Figure 4 and Figure S1, peaks consistent with free form of ARP (Retention time: 9.8 min, Fragment ions: [70.10]+, [192.20]+, [140.20]+) were resolved and detected in the sample obtained by the hydrolysis of carbonylated HEL and BSA, also confirming that ARP formation is more pronounced in HEL than BSA by comparing peak areas and ion intensities obtained under the same conditions of LC-MS/MS analysis. Meanwhile, no elution of peaks consistent with the PEN standard was observed from the hydrolysates of both proteins.

**Figure 5:**
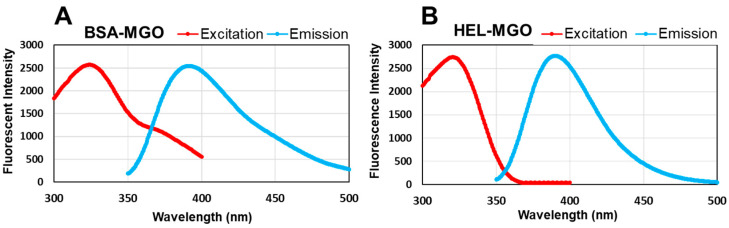
The fluorescence wavelengths of the AGEs produced by the reaction between MGO and BSA or HEL. After reaction with MGO for 7 days, fluorescence was measured in the range from 200 to 400 nm for excitation and 300 to 500 nm for emission. (**A**) 1 mg/mL BSA with 0.5 mM MGO; (**B**) 1 mg/mL HEL with 0.5 mM MGO.

To optimize the conditions, the excitation and emission wavelengths of fluorescent AGEs produced in the protein by the reaction of MGO with BSA and HEL were accurately measured. As shown in Figure 5, in this reaction system, the maximum fluorescence was obtained at Ex: 320 nm and Em: 390 nm for both BSA and HEL.

**Figure 6:**
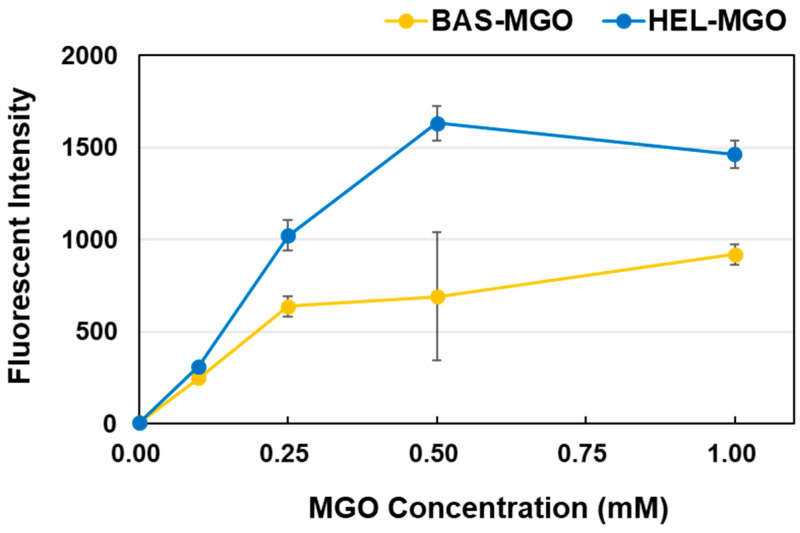
Effect of MGO concentration on the fluorescence intensity in the HEL–MGO and BSA–MGO reactions at the optimum wavelength. The fluorescence intensity at 390 nm for HEL–MGO and BSA–MGO was determined after 1 week of reaction at various concentrations of MGO.

A detailed comparison of the fluorescence intensity of HEL–MGO and BSA–MGO at the optimum wavelengths (Ex: 320 nm, Em: 390 nm) revealed that although both increased in a concentration-dependent manner with respect to MGO (Figure 6), the fluorescence intensity after a 1-week reaction was more than twice that of HEL–MGO than BSA–MGO. From these results, we considered that the optimum concentrations of MGO and HEL were 0.5 mM and 1.0 mg/mL, respectively.

Additionally, to optimize the measurement of the formation of fluorescent AGEs, the HEL–MGO reaction was performed at pH 7.4 (PBS) and 37 °C because it is appropriate to evaluate the antiglycation activity under physiological conditions.

**Figure 7:**
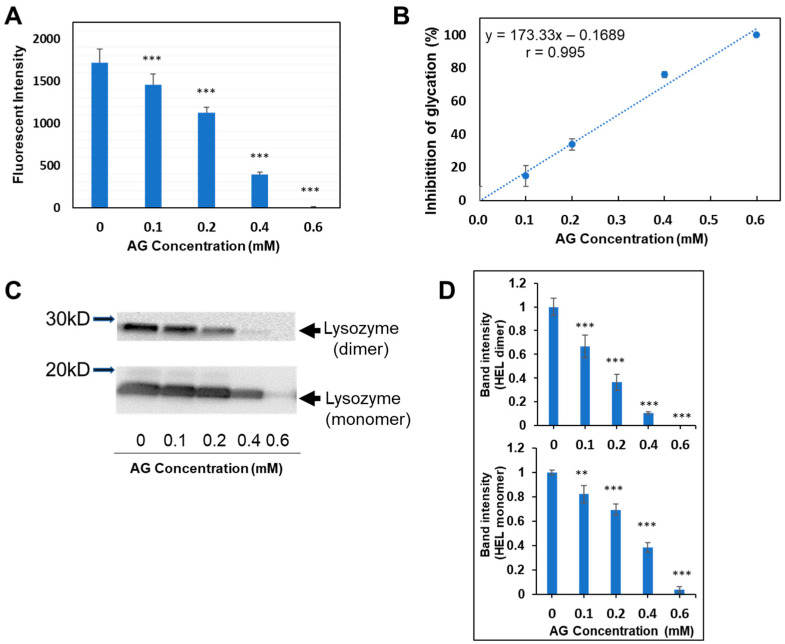
Evaluation of aminoguanidine using the HEL–MGO assay. The HEL–MGO method established in this study was applied to AG, a representative antiglycation compound. (**A**) Changes in the fluorescence intensity were dependent on the AG concentration. *** *p* < 0.001. (**B**) Regression line representing the relationship between AG concentration and inhibition rate (%). (**C**) Western blotting analysis of ARP-modified HEL monomer (14.3 kD) and dimer (28.6 kD), showing dependency on the AG concentration. (**D**) Densitometric analysis for the total band intensities of staining by anti-ARP antibodies shown in (**C**). Upper graph; HEL dimer, Lower graph; HEL monomer. ** *p* < 0.01, *** *p* < 0.001 versus 0 mM (Control without AG).

When the HEL–MGO method constructed in this study was applied to aminoguanidine (AG), a typical antiglycation compound, the fluorescence intensity at 390 nm decreased in manner dependent on an AG concentration (Figure 7A). The inhibition rate was calculated from the change in fluorescence intensity and graphed, yielding a concentration-dependent linear relationship with a high correlation coefficient (r = 0.995) (Figure 7B), indicating that this method was suitable for quantitative evaluation. The results also show that the MGO–HEL method identifies significant antiglycation activity at concentrations of 0.1 mM or higher for AG (Figure 7A). Furthermore, the precision of the HEL–MGO assay was examined; the intra-day variability of the antiglycation activity exhibited by AG was 0.35% to 8.59% (CV value; N = 4) in the range of 0.1 mM to 0.6 mM (Figure 7B). When the same samples were analyzed by western blotting, the band intensity of staining from anti-ARP antibodies also decreased in manner dependent on AG concentration (Figure 7C,D). This indicates that the decrease in fluorescence intensity clearly reflects the inhibition of ARP formation in lysozymes.

**Figure 8:**
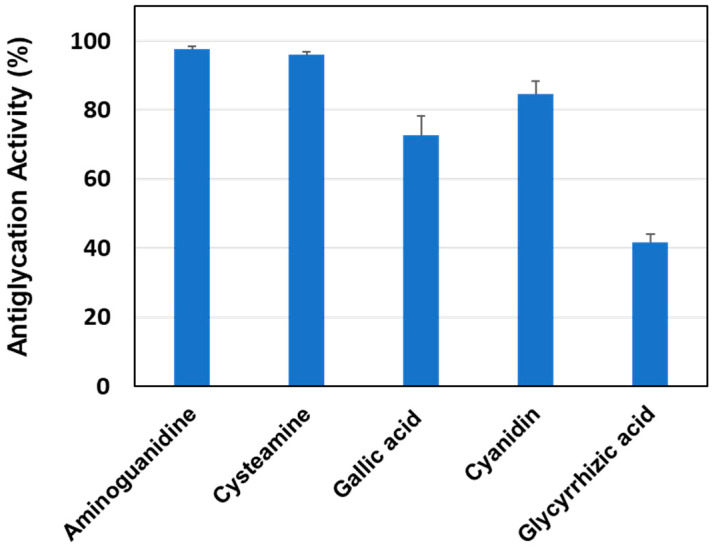
Antiglycation activities of five compounds known to react directly with MGO. The antiglycation activity of five compounds (0.5 mM) was determined by the HEL–MGO fluorescence assay using 1 mg/mL HEL and 0.5 mM MGO.

To demonstrate the practicality of this new evaluation method, the developed HEL–MGO fluorescence method was used to evaluate the antiglycation activity of five compounds known to inhibit glycation. The fluorescence intensity obtained for the evaluation of each compound was significantly lower than that obtained in the reaction between lysozyme and MGO in the absence of the evaluated compound, providing evidence of the antiglycation activity of the evaluated compounds. Figure 8 shows the anti-glycation activity (%) of each compound calculated from the decrease in fluorescence intensity using the previously mentioned Equation (1).

## 4. Discussion

In recent years, it has been suggested that AGEs are associated with cardiovascular disorders such as myocardial infarction and atherosclerosis in addition to diabetic angiopathy [28], neurodegenerative diseases such as Alzheimer’s disease, Parkinson’s disease, and amyotrophic lateral sclerosis [29], brain and liver damage caused by alcoholism [30], diabetic complications such as diabetic nephropathy, retinopathy, and neuropathy [31], bone metabolism abnormalities such as osteoporosis [32], aging [33], insulin resistance [34], and tumor growth and metastasis [35]. In addition, the effects of MGO, a metabolite of glucose produced in vivo, in various diseases has recently attracted attention, and our previous studies have also suggested that the glycation of specific proteins by MGO may cause disease [36].

Fluorescence assays using BSA have been widely implemented as a simple evaluation method for antiglycation activity. In these methods, an inhibitory effect on the formation of fluorescent AGEs, which increases following the Maillard reaction of BSA with glucose, is quantified as an antiglycation activity through the measurement of the decrease in fluorescence intensity. However, as a screening method for antiglycation compounds, there are many drawbacks: a long assay time (2–4 weeks), insufficient sensitivity, and it does not reveal the structure of the fluorescent AGEs produced [22].

In this study, we focused on the formation of fluorescent AGEs by several proteins other than BSA that have been reported to undergo glycation [37,38,39,40,41]. Using MGO as a glycation agent expected to have higher reactivity than glucose, we attempted to establish a method for evaluating the activity of antiglycation compounds based on the formation of ARP, a representative fluorescent AGE, and the inhibition of its formation. Compared with assays using BSA, the strongly fluorescent ARP-modified HEL was produced more quickly when HEL was used in this assay. HEL is a small basic protein containing 11 arginine residues (8.5%) with a molecular weight of 14.3 kD. The findings of the present study indicate that arginine residues of lysozyme are easily converted to the ARP structure when HEL is reacted with MGO. On the other hand, although the increase in fluorescence intensity of immunoglobulin due to reaction with MGO was negligible (Figure 2), Western blot analysis revealed that marked multimerization as well as the formation of ARP structures in the molecule occurred (Figure 3). The reason for this may be that in immunoglobulins, the detection of ARP-derived fluorescence is interfered with by structural changes associated with the formation of more complicated AGEs.

For the HEL–MGO (0.5 mM) reaction, the fluorescence intensity after 1 week was more than 10-fold higher than that from the BSA–glucose (50 mM) reaction and approximately 2.5-fold higher than that from the BSA–MGO (0.5 mM) reaction. It is known that Maillard reaction with glucose produces various fluorescent substances, such as vesperlysines [42], from BSA; however, for HEL, ARP was the main fluorescent AGE produced during the reaction with MGO. In the evaluation of aminoguanidine (AG), a typical antiglycation compound, the decrease in fluorescence at low concentrations, i.e., the inhibition of glycation, was clear and precise. Consequently, our established method can be considered useful as a screening method in terms of sensitivity, quantitativity and efficiency.

MGO, a reactive α-dicalbonyl as well as a precursor of AGEs increase the generation of reactive oxygen species [43,44,45]. Thus, antioxidant suppressed MGO-induced oxidative stress [46,47] and inhibit the oxidation of amino acids in protein, resulting AGEs formation [48]. Although MGO is a metabolite of glucose and its concentration in biological tissues is low, it is one of the carbonyl compounds in vivo, and its involvement in various diseases has recently been reported due to its high reactivity [49]. Therefore, compounds with activity to inhibit protein glycation by MGO or to break down MGO-induced AGEs are attracting attention [50].

From previous studies, the cellular concentration of MGO ranges from 1 to 5 μM and, in extreme cases, measurements of >300 μM have been identified [51]. Meanwhile, the condition study of this evaluation method showed that when 0.5 mM MGO was used, good sensitivity and accuracy were obtained in the measurement of fluorescence intensity. Namely, this evaluation method is an in vitro model system for antiglycation reactions and, like previous methods using BSA and glucose, deviates from physiological conditions with respect to protein and MGO concentrations. Thus, if this first screening method is used and the evaluated compound is found to exhibit antiglycation activity in the presence of high concentrations of MGO, the next step would be to examine its dose-dependent activity against lower concentrations of MGO, thereby demonstrating its usefulness under physiological conditions.

Since this method measures the fluorescence of ARP structures in HEL-MGO produced during glycation, overlap or interference with the intrinsic fluorescence of test compounds or contaminants may interfere with the method. In fact, pyridoxamine, which is the target for evaluation of antiglycolytic activity, is itself fluorescent, which poses a problem to be considered in the measurement of fluorescent AGEs. In addition, potential byproducts resulting from dicarbonyl compounds may interfere with this evaluation method. For example, melanoidin, a product other than AGEs produced in the Maillard reaction, has been reported to have fluorescent properties [52], and its interference cannot be ignored. Practically, if the coexisting interfering substances are trace amounts, this problem may be solved by subtracting the fluorescence intensity of the blank, excluding the lysozyme. However, this method was developed for use with somewhat purified natural products and synthetic compounds, and it is not suitable for crude samples such as plant extracts. Therefore, further improvement of the method is considered necessary when the test compound itself or a byproduct of the reaction with MGO emits strong fluorescence that overlaps with the wavelength of the ARP.

AGEs are formed through oxidative reactions by reactive carbonyl compounds such as MGO. At the same time, the mechanism by which antioxidants inhibit the formation of AGEs has been clarified [48]. Furthermore, a number of compounds with both antioxidant and antiglycation properties have been reported in recent years [11,53,54,55,56]. The present method can be used to evaluate not only the antiglycation activity, but also the antioxidant capacity to inhibit MGO-induced oxidative stress. Therefore, this method is also expected to be applied to the study of the biological activity of redox-active natural products. There needs to be more results obtained by another evaluation method to determine if carbonylation is inhibited by directly reacting with MGO or by indirectly inhibiting the reaction between the protein and MGO. However, the presence or absence of a direct reaction with MGO could be examined by quantifying the decrease in MGO in a protein-free system [57].

Cysteamine [12], cyanisidine [11], gallic acid [58], glycyrrhizic acid [59], and AG have been reported to act as antiglycation compounds [9]. We used them in this method as test compounds and verified their antiglycation activities. The present results confirm the antiglycation activity of polyphenols such as gallic acid, cyanidin, and glycyrrhizic acid, and the mechanism is assumed to be due to direct reaction with MGO as in the case of AG. However, if the polyphenols used in this study show inhibitory or protective effects against MGO-induced oxidative damage in cell culture systems, their antioxidant capacity is also expected to contribute.

The results of the present study reveal that MGO reacts with Arg residues of the protein at an early stage to form an ARP structure, inducing fluorescence. Although glucose and ribose produce pentosidine and other AGEs with stronger fluorescence, their Maillard reactions with proteins are complex and slow, making these reducing sugars unsuitable for a rapid screening method to evaluate compounds with weak antiglycation activity. The method developed in this study is superior in sensitivity and speed; thus, its future use to evaluate antiglycation activity should help to accelerate the discovery of new compounds for potential drug development.

## 5. Conclusions

The present study aimed to establish a novel in vitro screening method for antiglycation compounds including some antioxidants by using MGO as a glycation agent that reacts readily with amino groups in the protein. To find the fluorescent AGEs in carbonylated proteins with the strongest fluorescence that could be detected as early as possible, we performed a detailed comparison of the fluorescent AGEs formed from the reaction of MGO and various proteins, from which we identified HEL as the optimal protein. Subsequently, we established a novel evaluation method by determining the optimal conditions for the formation and detection of the fluorescent AGEs produced in the reaction between HEL and MGO. We then measured the activities of some antiglycation compounds by this novel method and confirmed our measurements using established methods. Thus, our present method is expected to be useful for screening compounds with antiglycation activity.

## Data Availability

The data are contained within the article and Supplementary Material.

## Supplementary Materials

The following supporting information can be downloaded at: https://www.mdpi.com/article/10.3390/antiox12122030/s1, Figure S1: Comparison of fragment ions obtained from the peaks at 9.8 min. (A) ARP standard solution, (B) Hydrolysate of HEL reacted with MGO for 7days, (C) Hydrolysate of BSA reacted with MGO for 7days.

## Abbreviations

AGEs, advanced glycation end products; ARP, argpyrimidine; BSA, bovine serum albumin; HEL, hen egg lysozyme; LC–MS/MS, liquid chromatography–tandem mass spectrometry; MGO, methylglyoxal; PEN, pentosidine; SDS–PAGE, sodium dodecyl sulfate-polyacrylamide gel electrophoresis.
