# Supplementary material for: Novel Fluorometric Assay of Antiglycation Activity Based on Methylglyoxal-Induced Protein Carbonylation
Source: Antioxidants (Basel). 2023 Nov 22;12(12):2030. doi: 10.3390/antiox12122030 (PMC10740428; doi:10.3390/antiox12122030)
Supplement: Supplementary file 1 [file antioxidants-12-02030-s001.zip › antioxidants-2713633-supplementary.pdf]

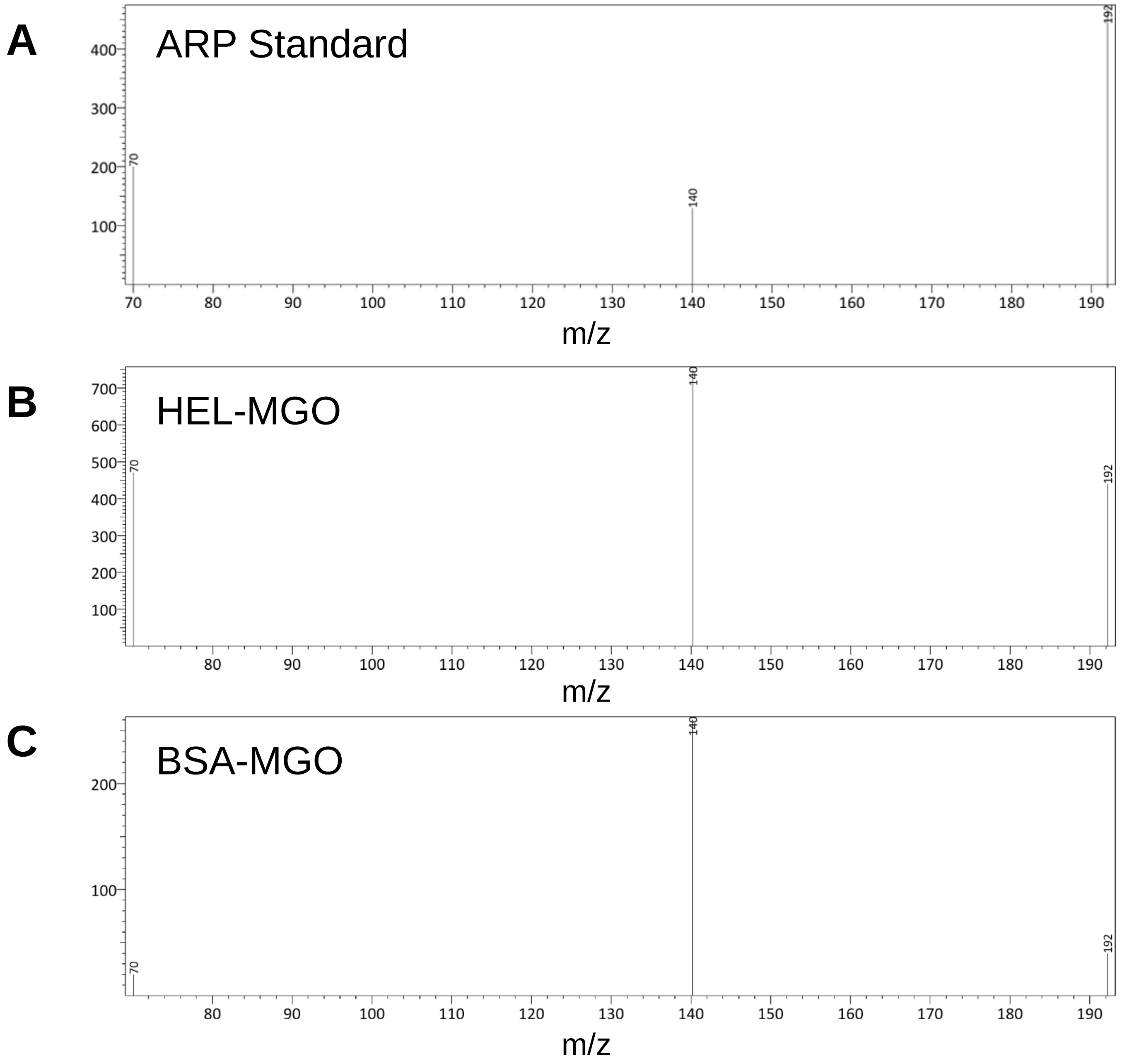

A
ARP Standard
400
300
200
100
70
140
192
70
80
90
100
110
120
130
140
150
160
170
180
190
m/z
B
HEL-MGO
700
600
500
400
300
200
100
70
140
192
80
90
100
110
120
130
140
150
160
170
180
190
m/z
C
BSA-MGO
200
100
70
140
192
80
90
100
110
120
130
140
150
160
170
180
190
m/z

Figure S1: Comparison of fragment ions obtained from the peaks at 9.8 min. (A) ARP standard solution, (B) Hydrolysate of HEL reacted with MGO for 7days, (C) Hydrolysate of BSA reacted with MGO for 7days.
